# A Jaw-Dropping Consequence: Nintedanib’s Role in Osteonecrosis of the Jaw

**DOI:** 10.7759/cureus.65689

**Published:** 2024-07-29

**Authors:** Oshin Rai, Kaitlyn N Romero, Natalie Shaykh, Ricardo Caldas, Vanshika Tripathi, Rebekah M Padilla, Abhinav Karan, Hui Jun Guo, Rafik Jacob

**Affiliations:** 1 Internal Medicine, University of Florida College of Medicine – Jacksonville, Jacksonville, USA; 2 Diagnostic Radiology, University of Florida College of Medicine – Jacksonville, Jacksonville, USA; 3 Program for Adults with Intellectual and Developmental Disabilities, University of Florida College of Medicine – Jacksonville, Jacksonville, USA

**Keywords:** idiopathic pulmonary fibrosis and its comorbidities, ipf, osteonecrosis of the jaw, medication-related osteonecrosis of the jaw, nintedanib

## Abstract

Nintedanib, a tyrosine kinase inhibitor, is a cornerstone in the management of idiopathic pulmonary fibrosis through its anti-fibrotic effects; however, its impact on wound healing is less studied. We present a case of medication-related osteonecrosis of the jaw (MRONJ) following the initiation of nintedanib. The patient's presentation prompted a drug holiday of nintedanib, resulting in a marked improvement in her symptoms. MRONJ is a disease requiring a high index of suspicion, and the number of inciting medications continues to rise. Nintedanib, as an inhibitor of angiogenesis, may have contributed to poor wound healing following dental extraction, subsequently leading to MRONJ.

## Introduction

Nintedanib is a small molecule tyrosine kinase inhibitor that competitively binds to multiple factors such as vascular endothelial growth factor (VEGF), platelet-derived growth factor (PDGF), and fibroblast growth factor (FGF) [1,2]. Inhibition of these factors affects the pathways involved in angiogenesis and activation of fibroblasts. This anti-fibrotic effect can be beneficial in a chronic and progressive disease such as idiopathic pulmonary fibrosis (IPF), where there is a decline in lung function due to fibrosis. Many clinical trials have shown the efficacy of nintedanib in reducing the decline in lung function, measured by an annual rate of decline in forced vital capacity [3,4].

One of the most commonly reported side effects is diarrhea and transaminitis [2,4], with data emerging regarding other complications. As antifibrotic therapy is an essential cornerstone of management in patients with IPF, and indeed other fibrotic lung syndromes, we anticipate that as the use of nintedanib increases, so will an awareness of its adverse effect profile.

Most recently, nintedanib has also been studied in severe cases of COVID-19 for its usefulness in treating the fibrotic phase of acute respiratory distress syndrome (ARDS) [5]. Medication-related osteonecrosis of the jaw (MRONJ), as the name suggests, is a severe drug reaction that leads to the destruction of bone in the maxillofacial region. Here we describe an association between nintedanib and MRONJ, a debilitating adverse effect that it is essential for prescribers to be familiar with.

## Case presentation

An 80-year-old female with a past medical history of IPF and hypertension presented for right lower jaw pain radiating to the ear and neck. The pain had been gradually worsening over two months, beginning after the extraction of two teeth from her right lower jaw, not in the setting of an infection. The patient’s pain became progressively worse with increasing numbness of the skin overlying the mandibular region. Her home medications included nintedanib for IPF and losartan. She had been on nintedanib for five years. Notably, she did not have a history of diabetes mellitus, peripheral vascular disease, coronary artery disease, or cancer.

On presentation, the patient had unremarkable vital signs apart from hypertension to 159/91 mmHg. The patient had mild leukocytosis of 12.4x10^3^/µL (reference range: 4.0-10.0x10^3^/µL) and thrombocytosis of 455 (reference range: 150-450x10^3^/µL). A comprehensive metabolic panel had no abnormalities (Table 1). Physical exam was remarkable for right facial edema and erythema over the site of the mandible, with no crepitus or fluctuance. The right upper gums demonstrated erythema and edema at the site of teeth 28 and 29, which had been previously extracted, without evidence of purulent drainage.

**Table 1:** Initial laboratory findings

Complete Metabolic Panel	Value	Reference Range
Sodium	137	135-145 mmol/L
Potassium	4.1	3.3-4.6 mmol/L
Chloride	102	101-110 mmol/L
Carbon Dioxide	26	21-29 mmol/L
Urea Nitrogen	14	6-22 mg/dL
Creatinine	0.74	0.51-0.96 mg/dL
Blood Urea Nitrogen/Creatinine Ratio	18.2	6-22
Glucose	101	71-99 mg/dL
Calcium	9.9	8.6-10.0 md/dL
Total Protein	6.9	6.5-8.3 g/dL
Albumin	4.0	3.8-4.9 g/dL
Total Bilirubin	0.3	0.2-1.0 mg/dL
Alkaline Phosphatase	69	35-104 IU/L
Aspartate Transaminase	15	14-33 IU/L
Alanine Transaminase	11	10-42 IU/L
Anion Gap	12	4-16 mmol/L
Estimated Glomerular Filtration Rate	82	≥ 60 mL/min/1.73m^2^
Complete Blood Count and Differential		
White Blood Cell	12.4	4.0-10.0 x10^3^/µL
Red Blood Cell	4.36	4.0-5/2 x10^6^/µL
Hemoglobin	14.0	12.0-16.0 g/dL
Hematocrit	42.3	35.0-45.0 %
Mean Corpuscular Volume (MCV)	97.0	78.0-100.0 fl
Mean Corpuscular Hemoglobin (MCH)	33.0	26.0-34.0 pg
Mean corpuscular hemoglobin concentration (MCHC)	33.1	31.0-36.0 g/dL
Red Cell Distribution Width (RDW)	13.3	11.0-14.6%
Platelet Count	455	150-450x103/µL
Mean Platelet Volume (MPV)	11.8	9.5-12.2 fl
Neutrophil %	81.7	34-73%
Bands %	1	0-10%
Lymphs %	9.8	25-45%
Monocytes %	2	2-6%
Metamyelocytes %	0	≤ 0%
Myelocytes %	0	≤ 0%
Promyelocytes %	0	≤ 0%
Neutrophil Absolute	10.1	1.4-7.5x103/µL
Lymphocyte Absolute	1.2	0.7-3.1x103/µL
Monocytes Absolute	0.82	0.1-0.9x103/µL
Immature Granulocytes Absolute	0.01	≤ 0.0x103/µL

**Figure 1:**
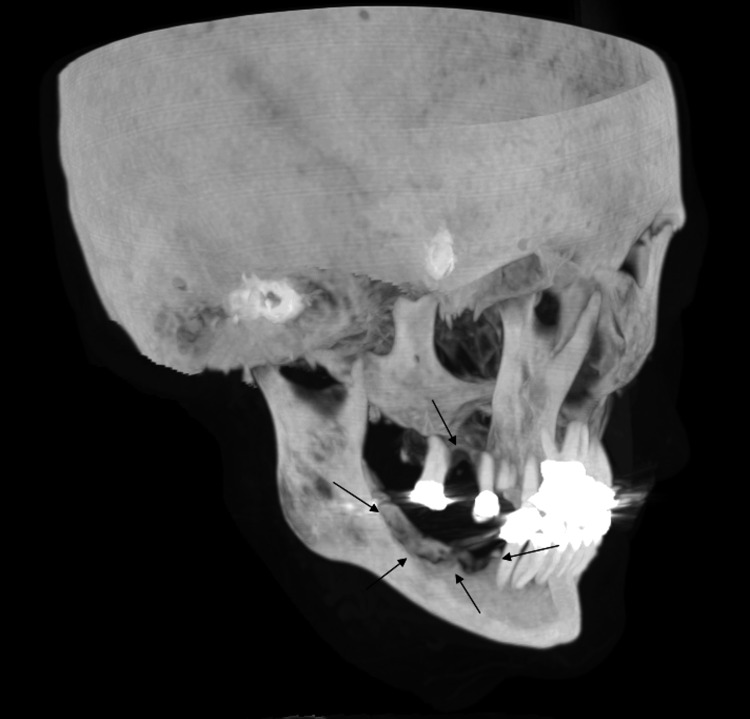
Three-dimensional volume rendered image demonstrates irregularity of the left mandible and maxilla representing osteonecrosis at prior tooth extraction sites (black arrows).

**Figure 2:**
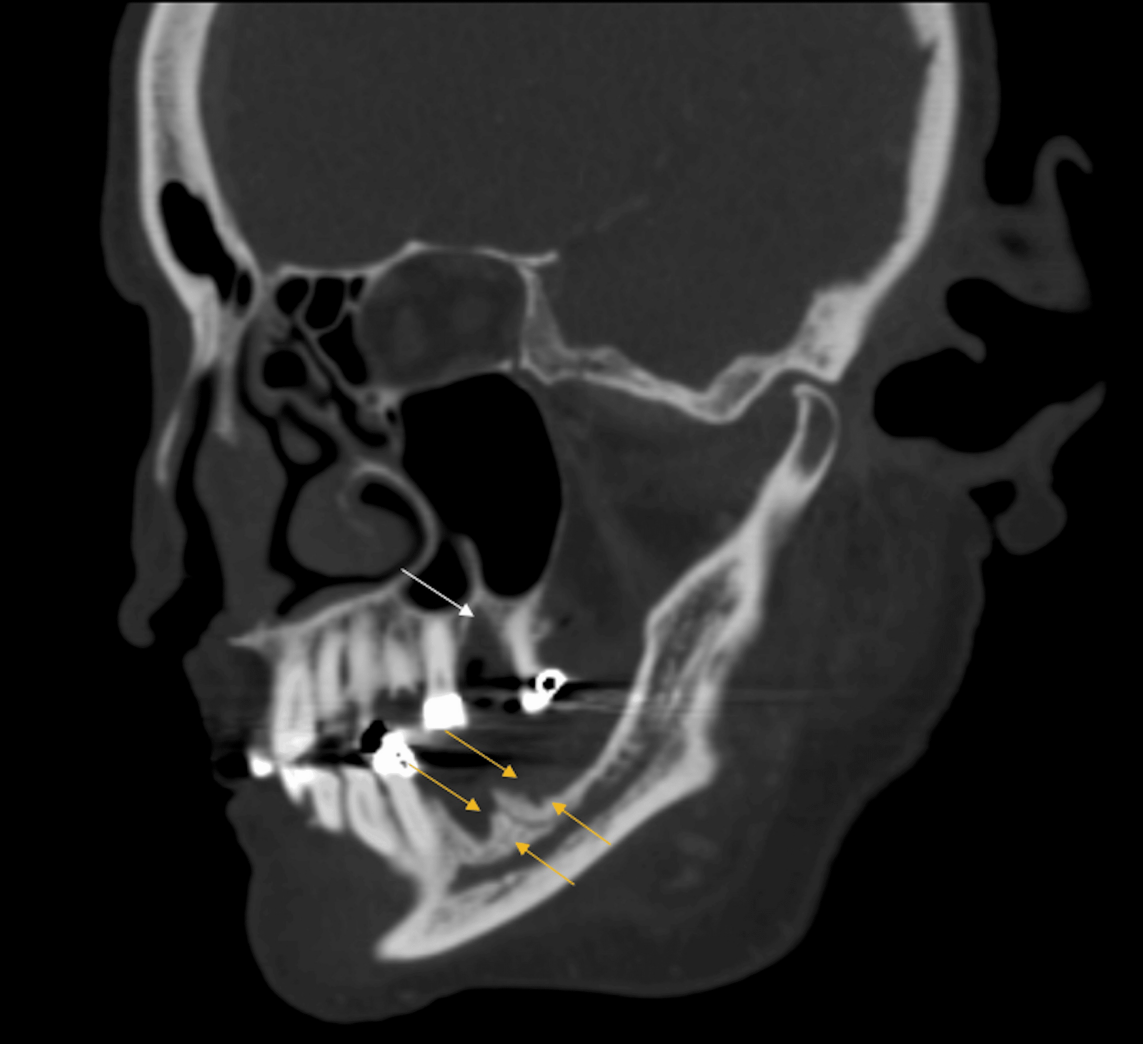
Oblique sagittal computed tomography reconstruction along the plane of the left mandible demonstrates mixed lytic and sclerotic lesions at prior tooth extraction sites of the maxilla (white arrow) and mandible (yellow arrow).

**Figure 3:**
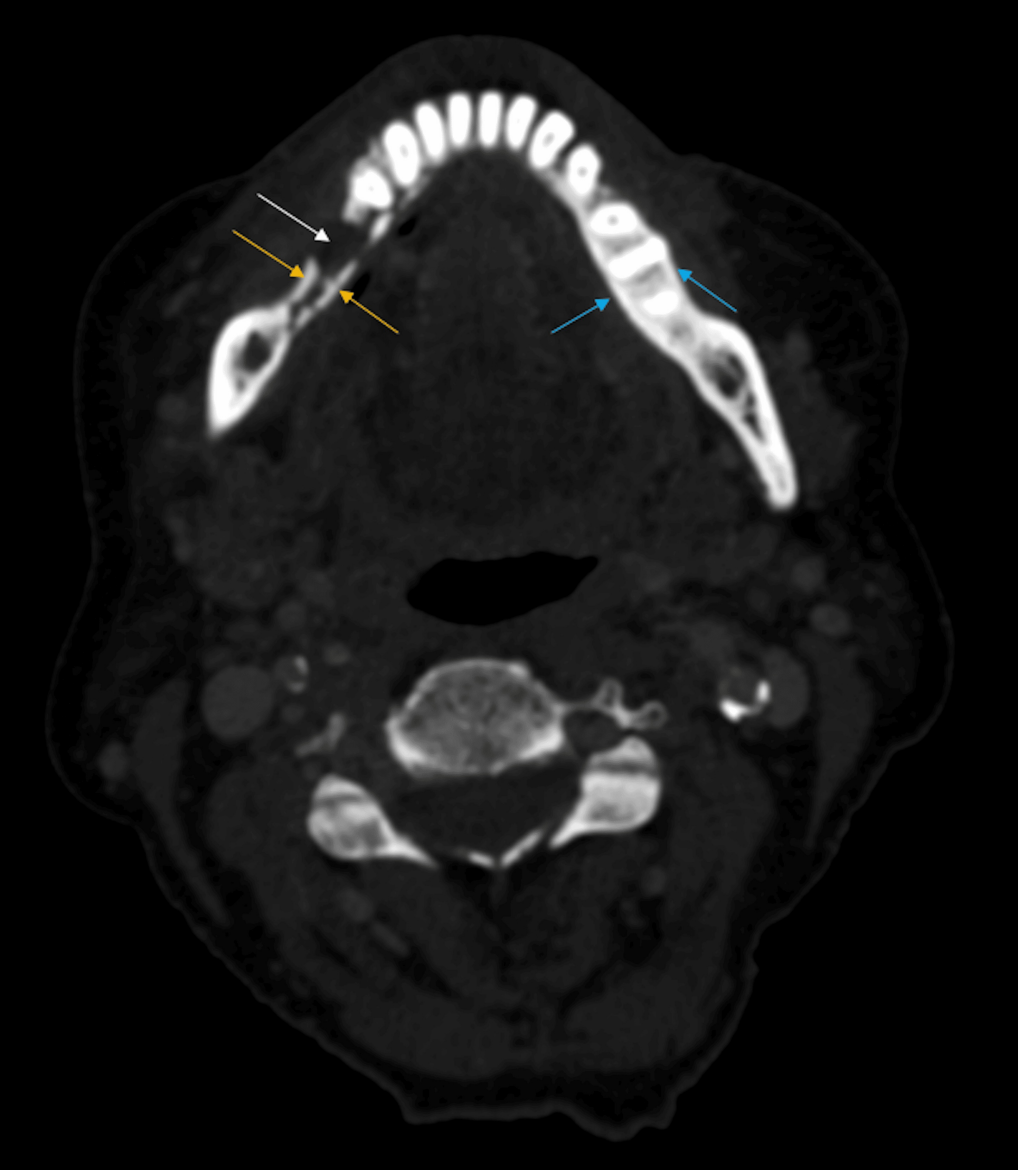
Axial computed tomography images of the mandible demonstrate a mixed lytic (white arrow) and sclerotic lesion (yellow arrows). Additional areas of contralateral sclerosis are also concerning for osteonecrosis (blue arrow)

A computed tomography (CT) scan of the maxillary and mandibular region was ordered to investigate the etiology of her underlying presentation, which demonstrated mixed lytic and sclerotic areas of the mandible and maxilla, consistent with osteonecrosis of the jaw (ONJ), alongside areas of previous tooth extractions that were concerning for non-healing wounds (Figures 1-3). The decision was made to discontinue the nintedanib due to ongoing ONJ and impaired healing in the setting of her prior dental extraction several months ago. The patient returned for a two-week follow-up visit with significant improvement in her jaw pain and significant improvement in her ONJ. 

## Discussion

In the context of wound healing, nintedanib plays a role in both the inflammatory and proliferative phase. Directly after an injury, the wound-healing process begins in the inflammatory phase. Nintedanib’s mechanism of inhibiting PDGF from platelets reduces the migration of fibroblasts, smooth muscle cells, macrophages, and proinflammatory cytokines such as interleukin-1 (IL-1) and tumor necrosis factor-alpha (TNF-α). This decline in macrophages results in a lower rate of cell apoptosis, which ultimately prohibits progression to further recruitment of fibroblasts and angiogenesis blunting the healing process. In the proliferative phase, PDGF serves to recruit fibroblasts that create collagen, which serves as the backbone of new tissue formation. Inhibition in this stage not only stunts tissue remodeling but also endothelial cells responsible for angiogenesis [6]. Without a properly stable tissue framework or arterial nutrient delivery, wound healing is difficult to facilitate. 

Osteonecrosis of the jaw is a result of cell death in the jaw, and if induced by medications is referred to as MRONJ. The pathophysiology of MRONJ is still poorly understood but has been described with bone-modifying agents (BMA), such as bisphosphonates and denosumab [7,8]. Bevacizumab, an antiangiogenic agent, when used as a monotherapy was shown to have a 0.2% risk of ONJ and when combined with BMA increased to 0.9%; however other studies showed no association between bevacizumab and MRONJ when used as a single agent [8]. Soutome et al. found that patients on BMA who had a tooth extraction did not have an increased risk of MRONJ, but instead infected teeth had an increased risk of MRONJ [7].

After the removal of two teeth, our patient was not able to facilitate wound healing, a process reliant on angiogenesis. Without being on a bisphosphonate, we propose our patient's progression to ONJ was a result of nintedanib therapy and its disruption of angiogenesis. She did not have a known history of poor oral hygiene at baseline, chronic inflammation, diabetes mellitus, ill-fitting dentures, and maxillary or mandibular bone surgery as other confounding risk factors. An association between nintedanib and MRONJ was first anecdotally recognized following the widespread use of nintedanib in certain countries as part of experimental therapy for lung fibrosis post COVID 19 [8]. Despite this, there exists very limited data on this association. AlRowis et al. mention that although drug holidays are one proposed solution, there is still no adequate evidence to support this [9]. In this patient who had no other apparent cause of MRONJ, nintedanib was presumed to be related to his underlying disease. Subsequently, nintedanib was discontinued, and she encountered a dramatic improvement in her symptoms, making it reasonable to conclude it is a benign and viable approach for the appropriate patient. 

## Conclusions

Nintedanib is indicated for its anti-fibrotic properties in IPF, but its dampening effects on the inflammatory response should be carefully considered. The ability for it to weaken tissue integrity should be thoroughly examined in patients with significant wounds. Further understanding of pathophysiology and drugs associated with MRONJ can help to develop better management strategies for this condition. While the association between nintedanib and MRONJ has not been studied in randomized controlled trials, it is evident that due to its increasing utility in fibrotic lung diseases, prescribers should remain aware of potential adverse effects including the possibility of MRONJ.
